# Lessons from irritable bowel syndrome: potential for understanding and managing post-COVID

**DOI:** 10.3389/fimmu.2026.1717324

**Published:** 2026-03-16

**Authors:** Andreas Stallmach, Peter Layer, Katrin Katzer, Philipp A. Reuken

**Affiliations:** 1 Department of Internal Medicine IV (Gastroenterology, Hepatology and Infectious Diseases), Jena University Hospital, Jena, Germany; 2 MEDIZINICUM, Hamburg, Hamburg, Germany

**Keywords:** irritable bowel syndrome, long-covid, post-COVID, postinfectious long-term sequelae, SARS-CoV-2

## Abstract

Post-COVID presents a complex medical challenge characterized by persistent symptoms following SARS-CoV-2 infection. Similarities between post-COVID and post-infectious Irritable Bowel Syndrome (PI-IBS) suggest that the latter can serve as a useful model for understanding pathophysiological mechanisms and developing therapeutic approaches. Both conditions are functional disorders triggered by an acute infection, with multifactorial etiology and limited biomarker-based diagnostics. The variability of symptoms and the high frequency of comorbidities make these disorders particularly difficult to diagnose. Diagnostic efforts may be further hindered by the stigmatization of such disorders among healthcare providers, the health insurance industry, and the general public. This article explores the parallels between PI-IBS and post-COVID, highlighting, on the one hand, what can be learned from the management of IBS to better address the needs of patients with post-COVID long-term sequelae, and, on the other hand, raising doubts—based on decades of research into drug therapy development for IBS—about the likelihood of a rapidly available treatment for post-COVID.

## Introduction

After the first cases of acute SARS-CoV-2 infection, a substantial proportion of patients were found to experience long-lasting sequalae (1, 2). The World Health Organization (WHO) defines symptoms that persist for 12 weeks or longer following an acute SARS-CoV-2 infection, without any other plausible explanation, as post-COVID-19 syndrome (PCS) (3).

While the initial waves of SARS-CoV-2 infection led to PCS in approximately 6–10% of cases (4), subsequent increases in population immunity and the emergence of less pathogenic variants have significantly reduced the likelihood of long-term sequelae (5). Even though the incidence of PCS is decreasing, patients with long-term courses following infections during the first waves, as well as newly affected individuals, continue to present a challenge in medical care.

One major challenge in handling of PCS patients is the broad spectrum of symptoms. Until today, more than 200 different symptoms have been attributed as long-term sequelae of a SARS-CoV-2 infection (6) with fatigue being one of the most frequently reported symptoms (7). Additionally, anxiety, depression, cognitive problems and respiratory symptoms are often reported (8). In addition, an increased incidence of functional gastrointestinal disorders, including irritable bowel syndrome (IBS), functional diarrhea, functional constipation, and functional dyspepsia, was observed in COVID-19 patients compared to healthy controls at a six-month follow-up (9). Furthermore, a large-scale, multicenter, controlled study involving over 2,000 hospitalized patients demonstrated that individuals with SARS-CoV-2 infection exhibited significantly higher rates of IBS—characterized by abdominal pain and altered bowel habits—than non-COVID patients (3.2% vs. 0.5%, respectively) (10).

PCS and post-infectious IBS represent sequelae of infectious diseases in which the acute phase resolves before persistent symptoms emerge. Both conditions are characterized by ongoing immune activation with low-grade inflammation, alterations of the intestinal microbiome, and disturbances of the gut–brain axis and autonomic regulation. They can thus be conceptualized as post-infectious long-term syndromes arising from the interplay of immune dysregulation, microbial imbalance, and neurovegetative dysfunction. While post-COVID manifests as a systemic disorder, post-infectious irritable bowel syndrome is primarily confined to the gastrointestinal tract. Due to its clinical manifestations, IBS is considered also a potentially disabling condition. It is associated with substantial healthcare expenditures, reduced occupational productivity and school attendance, and a significantly impaired health-related quality of life among affected individuals (11, 12). Despite its high prevalence in contemporary gastroenterological practice, the pathophysiological mechanisms underlying IBS remain incompletely understood. Similar to PCS it is recognized as a complex, multifactorial disorder influenced by a variety of factors including age, sex, genetic predisposition, dietary habits, psychosocial stressors, alterations in the gut microbiota, subclinical inflammation, and visceral hypersensitivity (13–15). The risk factors for the development of PCS and IBS are therefore the same — another indication of their numerous similarities. Both conditions are reported more frequently in female patients, younger age and can be associated with severity of the initial infection and psychological preconditions (7, 16–18).

Another major challenge for both, patients and physicians, is the absence of objective markers in laboratory tests or imaging diagnostics. The German PCS-guidelines emphasize that no routinely available parameters can confirm or exclude PCS (19). Furthermore, the lack of a universally accepted, coherent pathophysiological concept complicates the development of causal therapeutic approaches.

It is widely accepted for both conditions that no single, definitive cause exists (16, 20), making a monotherapy-based approach unlikely to cure the condition. Understanding IBS, which has been known for many decades, with the various research and treatment approaches can also provide valuable insights into effective diagnostic and therapeutic strategies for PCS patients. IBS — particularly in its post-infectious form (PI-IBS) — serves as a well-studied model of a functional, chronic disorder that arises following an acute infectious event. This article explores the parallels between PI-IBS and PCS, highlighting how management strategies developed for PI-IBS can inform and enhance the care of patients with PCS.

## Diagnostic challenges

Patient symptoms following COVID-19 and in individuals with PI-IBS often remain unexplained by objective findings in routine testing, highlighting the complexity and evolving nature of these conditions. Diagnostic strategies often depend on persistent symptom patterns and ruling out alternative causes, and should follow national and international guidelines or consensus recommendations (19, 21–24). Results are often normal in PCS or IBS patients; only through advanced methods of clinical research can morphological, immunological, or genetically based deviations from healthy individuals be detected. However, due to their low specificity, these findings are not suitable for use in routine clinical practice. Both conditions are marked by variable symptom expression. Recent studies suggest, that one such mechanism involves a reduction in tissue blood supply, which is associated with a proposed quantifiable index (25). This mechanism was evaluated by analyzing the incidence rates of Long COVID symptoms directly linked to this mechanism, and it was found to account for the majority (76%) of the reported symptoms (26). PI-IBS patients may present with abdominal pain, bloating, diarrhea, or constipation, in these patients Rome IV criteria guide diagnosis. PCS patients report fatigue, “brain fog”, chest pain, gastrointestinal symptoms, and more (7). This heterogeneity complicates diagnosis and management but underscores the need for personalized care (see also **Table 1**). It is important that if the diagnostic results are negative and there are no changes in symptoms or red flags (e.g. fever, anemia, weight loss, visible or occult blood in the stool), the test should not be repeated (“there must be something wrong, I feel so bad”). Attentive, empathetic care is important in this context. Healthcare professionals should communicate this uncertainty to patients, but at the same time also communicate that adequate diagnostics should rule out life-threatening diseases.

**Table 1:** Diagnostic tools and strategies in IBS.

Diagnostic strategy	Background
Clinical evaluation	Thorough history and physical examination to assess symptoms, their duration, and any associated factors. Fulfillment of the Rome IV-criteria as well as absence of red flags should be ascertained in all patients presenting with symptoms compatible with IBS.
Stool tests	1. Fecal calprotectin. 2. Occult blood testing (if malignant disease is deemed possible). 3. Microbiological testing (if gastrointestinal infection is deemed possible, especially in the diarrhea-predominent type).
Blood tests	Basic laboratory tests including inflammatory parameters (C-reactive protein (CRP) and white blood cell count (WBC)) as well as testing for celiac disease
Ileocolonoscopy/gastroscopy including biopsies	To rule out malignancy, inflammatory bowel disease (IBD), microscopic colitis, celiac disease or Whipple’s disease.
Breath tests	To rule out lactose or fructose malabsorption or small intestinal bacterial overgrowth (SIBO), if deemed possible.
Abdominal ultrasound	Should be performed in all patients to rule out other abdominal pathologies, e.g. tumors.
Imaging, e.g. CT or MRI	Should only be performed in specific cases, especially if red flags are present.
Psychological assessment	Evaluation of mental health conditions that may influence IBS symptoms, such as anxiety or depression.

## Pathophysiological parallels

### Post-infectious onset

**Figure 1:**
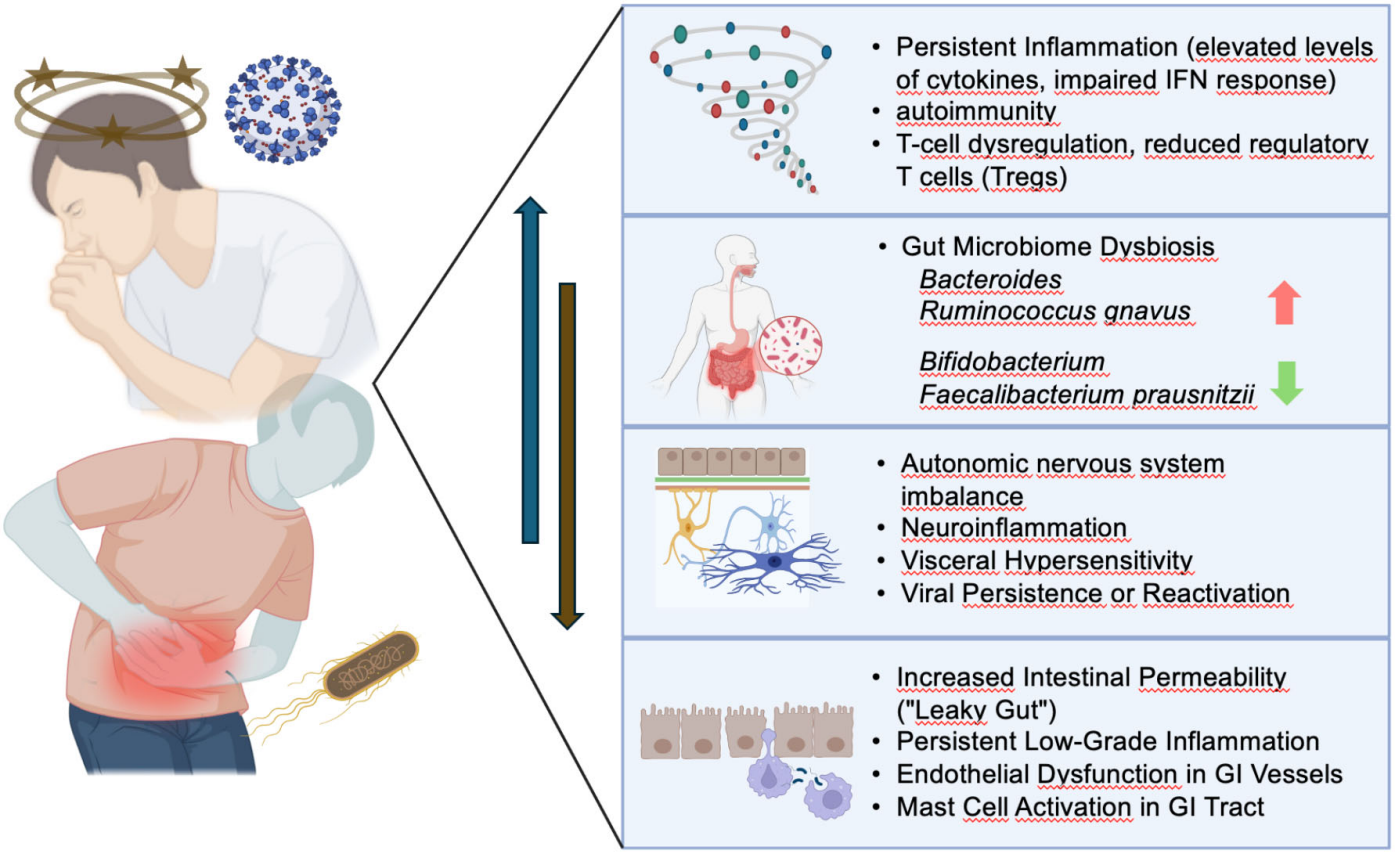
Pathogenetic similarities between PCS and PI-IBS. Systemic immunological imbalances are more pronounced in PCS (the blue arrow indicates their increasing relevance in PCS), whereas disturbances in the microbiota and gastrointestinal permeability (brown arrow) are likely of greater significance in PI-IBS.

Both PCS and PI-IBS frequently follow acute infections. In IBS, the onset often occurs after microbial gastroenteritis, whereas PCS follows viral infection. Timeline and features of symptom development in both cases suggest shared mechanisms of post-infectious sequelae. PI-IBS is defined by a constellation of persistent gastrointestinal symptoms that develop subsequent to an episode of infectious enteritis, with other causes of symptoms having been excluded. Common clinical features include abdominal pain, altered bowel habits—such as diarrhea, constipation, or a combination of both—bloating, and increased visceral sensitivity. The severity and duration of symptoms can vary considerably, often leading to substantial impairment in daily functioning and quality of life. Considerable efforts were made to elucidate the pathophysiological mechanisms underlying PI-IBS. Current evidence suggests that the inciting gastrointestinal infection induces deficient intestinal barrier functions, aberrant immune responses and disturbances of the composition of the intestinal microbiota. This, in turn, may result in chronic low-grade inflammation, altered gut motor and secretory functions, and visceral hypersensitivity (see also **Figure 1**).

These interconnected processes are thought to contribute to both the onset and persistence of PI-IBS symptoms. Epidemiological studies have investigated the incidence and prevalence of PI-IBS across diverse populations, underscoring its clinical relevance. Reported incidence rates following infectious enteritis range from 5% to 32%, depending on the characteristics of the study cohort and the duration of follow-up. PI-IBS is diagnosed when at least 6 months have passed since the initial infection and symptoms have been present for at least 3 months. In subsets of affected individuals, symptoms may improve over 6 to 24 months. However, in other patients, PI-IBS may persist for several years or even decades. Poor prognostic factors include: severe course of the initial infection, psychological stress, female sex, and pre-existing gastrointestinal conditions. As there is no causal therapy, treatment is symptom-oriented. A comparison between PI-IBS and PCS is provided in **Table 2**.

**Table 2:** Comparison of PI-IBS and PCS.

Parameter	PI-IBS	PCS
Trigger	*Campylobacter*, *Salmonella*, *Shigella*, *E. coli*, *Clostridioides difficile*, or viruses (e.g., norovirus)	SARS-CoV-2 virus
Time interval between infection and symptom onset	6 months (16)	3 months (3)
Long-term course	Spontaneous improvement in about 25-50% over several years (27–29), but chronic courses over years have also been described	Reduction of severe cases from 31.3 to 19.4% within six months (30) 39.6% of patients report inability to work (31)
Symptoms	Abdominal pain, bloating, altered bowel habits (diarrhea, constipation, or both)	Fatigue, dyspnea, cognitive dysfunction (“brain fog”), gastrointestinal complaints (e.g., nausea, diarrhea), and others

### Immune dysregulation

Both PCS and classical post-infectious IBS (PI-IBS) are characterized by persistent immune activation following an acute infectious insult (32, 33). They share several immunopathological features, including low-grade mucosal inflammation, increased intestinal permeability, dysbiosis, and heightened immune cell activity. Elevated levels of pro-inflammatory cytokines such as IL-6, IL-8, TNF-α, and IL-1β have been documented in both conditions, contributing to chronic gastrointestinal symptoms and altered gut-brain axis signaling (34–36). In PI-IBS, the immune response is typically localized to the gastrointestinal tract and follows a clearly defined episode of bacterial or parasitic gastroenteritis. Histological studies have shown increased intraepithelial lymphocytes and mucosal mast cells, particularly in proximity to enteric neurons, suggesting a neuroimmune component underlying the characteristic visceral hypersensitivity. In contrast, PCS-IBS arises in the aftermath of SARS-CoV-2 infection and can occur even in patients who did not experience acute gastrointestinal symptoms. SARS-CoV-2 infects intestinal epithelial cells via ACE2 receptors, and viral RNA and proteins have been detected in the intestinal mucosa for weeks to months after the resolution of respiratory symptoms, indicating potential viral persistence and chronic immune stimulation (37). This has been associated with persistent elevation of systemic inflammatory markers and ongoing immune dysregulation, a hallmark of PCS (38). Moreover, PCS-IBS differs from PI-IBS in its systemic immune involvement. While PI-IBS is generally confined to the gut, PCS-IBS may reflect a broader immune dysregulation affecting multiple organ systems. Several studies have reported T cell exhaustion, altered interferon signaling, and the presence of autoantibodies in individuals with PCS, suggesting overlapping autoimmune features (39). These immune phenomena may influence gastrointestinal function through complex systemic and local mechanisms that are not typically observed in classical PI-IBS. In summary, while both PI-IBS and PCS-IBS share common features of mucosal immune activation, the latter is distinguished by more widespread and prolonged immune dysregulation. PCS-IBS may represent a distinct clinical phenotype within the spectrum of post-infectious functional gastrointestinal disorders, influenced by the unique virological and immunological properties of SARS-CoV-2. An overview of similarities and differences regarding immune dysregulation is provided in **Table 3**.

**Table 3:** Similarities and differences between PSC and PI-IBS regarding immune dysfunction.

Mechanism	PCS	PI-IBS
Similarities		
Low-grade mucosal inflammation	present (40, 41)	
Increased intestinal permeability (“leaky gut”)	Observed (42, 43)	
Elevated pro-inflammatory cytokines	IL-1β, IL-6, IL-8, TNF- α (44–48)	
T-cell activation	present (49, 50)	
Mast cell involvement	Possibly role in pathophysiology (51, 52)	Established role in IBS pathophysiology (53)
Differences		
Systemic inflammation	More prominent — COVID can trigger widespread immune dysregulation and “cytokine storm” in acute phase (54)	Mostly confined to the gut mucosa (55)
Viral immune signature	Associated with type I interferon response and viral persistence (56, 57)	Often driven by bacterial toxins or pathogen-associated molecular patterns (PAMPs) (58, 59)
Immune exhaustion/dysregulation	Evidence of T-cell exhaustion and long-term immune dysfunction in some PCS cases (49)	Less documented; immune activation is more localized and self-limited (53)
Elevated cytokines	tachykinins, IFN- α 2, IL-10, IL-32 (46, 47, 56)	IL-4, IL-5, IL-17, IL-22 (48, 60–62)

### Gut microbiota and dysbiosis

Gastrointestinal dysbiosis—an imbalance in the composition, diversity, or metabolic function of the gut microbiota—has been implicated in both PCS and IBS, suggesting a possible shared pathophysiological pathway. However, while overlaps exist, the causal relationships and underlying mechanisms differ in nuance and extent.

Recent longitudinal and cross-sectional studies have demonstrated that individuals with PCS often exhibit persistent alterations in their gut microbiota following acute SARS-CoV-2 infection (63–65). PCS is significantly associated with persistent intestinal dysbiosis, characterized by a marked reduction in microbial diversity and the depletion of beneficial commensal bacteria (63, 66). Specifically, patients experiencing lingering symptoms often exhibit a significant loss of short-chain fatty acid (SCFA)-producing taxa, such as *Fecalibacterium prausnitzii, Eubacterium rectale*, *Akkermansia and Bifidobacterium pseudocatenulatum*, which are critical for maintaining the intestinal barrier and regulating systemic inflammation (63, 67–69). Conversely, there is an enrichment of opportunistic pathogens and pro-inflammatory species, including *Ruminococcus gnavus* and *Bacteroides vulgatus*, which have been correlated with persistent fatigue and neuropsychiatric symptoms (63, 70). Beyond bacterial dysbiosis, some studies have also provided insights into the associations between fungal dysbiosis including gut candidiasis, and PCS (71). This state of dysbiosis is thought to facilitate microbial translocation—the “leaky gut” phenomenon—whereby microbial products enter the bloodstream, sustaining low-grade systemic inflammation and potentially driving the multi-organ pathology observed in post-COVID syndrome through the gut-lung and gut-brain axes (66, 70). Although definitive proof of causality in humans remains challenging, the temporal sequence of SARS-CoV-2 infection, followed by persistent dysbiosis and long-term symptoms, supports a causal hypothesis.

In IBS, dysbiosis has long been implicated as one of several interrelated pathophysiological mechanisms (72, 73). Studies consistently report altered gut microbial composition in IBS patients, including reduced microbial diversity, decreased levels of short-chain fatty acid (SCFA)-producing bacteria, and an increase in gas-producing or pro-inflammatory microbes (74). Importantly, PI-IBS provides the strongest evidence of dysbiosis as a potential causal factor, much like in PCS gastrointestinal sequelae (**Table 4**).

**Table 4:** Similarities and differences of intestinal dysbiosis in PCS and PI-IBS.

Parameter	PCS	PI-IBS
Similarities		
Microbiota Signatures	loss of beneficial commensals and an overrepresentation of pro-inflammatory or pathogenic species	
Neuroimmune Pathways	Dysbiosis may contribute to symptomatology via interactions with the gut-brain axis	
Differences		
Causality Strength	Emerging evidence for dysbiosis as a *driver* for some symptoms (64, 75)	Dysbiosis is *associated* but not always primary cause (76)
Systemic Manifestations	Multisystemic (neurological, cardiovascular, etc.)	Primarily gastrointestinal
Therapeutic Trials	Early-stage for microbiota-directed therapies	Ongoing, including probiotics, FMT, antibiotics

In conclusion, both PCS and PI-IBS involve gut dysbiosis, but differ in their clinical scope and strength of causal evidence. While dysbiosis in PI-IBS is one of several contributors to a complex functional disorder, PCS dysbiosis may act as a downstream consequence of SARS-CoV-2 infection that actively contributes to ongoing systemic symptoms. It is important to note that the alterations reported across various PCS and PI-IBS studies differ considerably, potentially reflecting the influence of dietary, lifestyle, environmental, and genetic factors on gut microbiome composition. Furthermore, as the majority of microbiome samples were collected several months after symptom onset, secondary effects cannot be ruled out. Consequently, experimental models and early interventional studies employing microbiota-modulating therapies are essential to establish causality.

### Neuro-immune and autonomic dysfunction

Both disorders involve dysfunction in the autonomic nervous system and neuroimmune communication. Patients with IBS and PCS may exhibit symptoms of postural orthostatic tachycardia syndrome (POTS), abnormal heart rate variability, and heightened visceral or systemic hypersensitivity. Systemic inflammation and neuroinflammation have been implicated in the development of depressive disorders and other neurocognitive symptoms including fatigue following COVID-19 (77–79). It is now widely believed that immune system dysregulation and ongoing inflammatory signaling play a central role in the pathophysiology of many PCS symptoms by disrupting multiple homeostatic systems (80, 81). Disrupted catecholamine and acetylcholine signaling may lead to autonomic dysfunction, manifesting as tachycardia, impaired regulation of vascular tone and coagulation, and a reduced capacity to control inflammation (82).

Post-exertional malaise (PEM) is frequently observed in PCS alongside fatigue and marked exercise intolerance (7, 30). Although these symptoms are pathophysiologically related, they represent distinct clinical phenomena within the spectrum of PCS manifestations. Exercise intolerance is characterized by an inability to sustain physical exertion due to diminished energy reserves or the rapid onset of symptoms such as palpitations, tachycardia, or dyspnea. In contrast, PEM refers to a delayed and maladaptive physiological response following exertion, leading to the exacerbation of symptoms including fatigue, pain, cognitive dysfunction, and a reduction in overall physical functioning (20). PEM can be triggered not just by exercise, but also by moderate physical activity or everyday tasks (83, 84). In individuals with severe myalgic encephalomyelitis/chronic fatigue syndrome (ME/CFS), even minimal exertion—such as sitting upright or performing oral hygiene—may provoke PEM.

Although there are hardly any systemic manifestations in IBS compared to PCS reported, there are still indications of a dysregulation of the autonomic nervous system, with reports showing deterioration of parasympathetic response and increase in sympathetic response when performing autonomous neuropathy tests (e.g. response to standing up, deep breathing, Valsalva) (85–87). Indeed there is evidence suggesting a greater prevalence of impaired parasympathetic function in constipation-predominant IBS compared to other IBS subgroups (88).

## Therapeutic insights from IBS management

A key lesson from IBS is the effectiveness of a multimodal treatment approach, including dietary interventions, psychological support, and pharmacological management. Applying similar principles to PCS may offer symptom relief and improve functional outcomes. Low-FODMAP diets, probiotics, and other gut-targeted nutritional therapies have shown benefit in IBS and may hold promise for PCS patients with gastrointestinal involvement or microbiome alterations. A recent phase III study indeed reported an improvement in PCS related fatigue and tissue metabolism using a 3-month course of a symbiotic mixture (89) and further studies are recruiting (90).

## Symptomatic treatment of PCS and IBS: commonalities and differences

PCS and PI-IBS are both chronic, multisystem disorders characterized by heterogeneous and often overlapping symptomatology. To date, no definitive causal pharmacological therapies have been established for either condition. As such, the cornerstone of clinical management remains symptomatic treatment, which, while sharing certain similarities across both syndromes, also diverges in important ways due to their differing pathophysiological underpinnings. Both PCS and IBS are marked by non-specific, fluctuating symptoms—including fatigue, pain, and gastrointestinal disturbances—that necessitate individualized, multimodal therapeutic approaches tailored to the patient’s symptom profile and functional status. Dietary management is central in PI-IBS and is increasingly recognized in PCS management. For IBS, the low-FODMAP diet is often effective in reducing bloating and abdominal discomfort (91). For PCS, diet and nutrition may support recovery by targeting inflammation and the gut microbiome to alleviate symptoms and improve functional outcomes (92). In a preliminary study, a combined dietary strategy beginning with a ketogenic diet to reduce inflammation and oxidative stress, followed by the Mediterranean diet rich in whole grains, olive oil, fruits, vegetables, legumes, nuts, and lean proteins, has been proposed to improve metabolic health and alleviate symptoms, especially in individuals with obesity (93). Nutritional supplementation with compounds like acetyl L-carnitine, hydroxytyrosol, and vitamins B, C, and D has shown potential benefits in reducing fatigue and supporting immune recovery in small pilot studies (94). Additionally, dietary polysaccharides were found to provide immunomodulatory, antioxidant, and prebiotic effects in PCS and therefore may help modulate the immune response, although further validation is needed (95). While these interventions are promising, they are still experimental settings and evidence for nutritional intervention is still scare. Therefore, further research is required to establish optimized recommendations and to standardized dietary guidelines for PCS.

Cognitive behavioral therapy (CBT) and other psychological interventions are recommended for both IBS and PCS, particularly due to the strong biopsychosocial interplay. For IBS, CBT has demonstrated effectiveness in reducing symptom severity (96). In PCS, psychological support is emphasized for managing anxiety, depression, and fatigue (2).

Despite these similarities, key differences exist in the treatment strategies due to the systemic nature of PCS. Unlike PI-IBS, PCS often includes respiratory, neurological, and cardiovascular symptoms. Although these respiratory and neuro-psychological symptoms are reported more often than GI-Symptoms (7, 8) and some of these symptoms are linked to the GI-tract, there are also other pathophysiological aspects that are involved in the pathogenesis of PCS, such as a disrupted blood-brain barrier (97, 98), autoimmunity (20). This necessitates a broader therapeutic approach, involving pulmonary rehabilitation, pacing strategies for post-exertional malaise, and sometimes anticoagulants or antihistamines (99). IBS treatment, in contrast, is typically confined to the gastrointestinal tract and its functional regulation. While both conditions can be chronic, IBS is considered a lifelong functional disorder, whereas PCS is expected to resolve in some individuals over time. This distinction impacts the therapeutic goal—long-term symptom management in IBS versus potentially temporary support and monitoring in PCS.

## Patient-physician-interaction

Given both, the complex multisymptomatic presentation and the therapeutic options, in PSC and IBS, strong physician-patient communication becomes crucial to ensure accurate diagnosis, effective management, and appropriate psychosocial support. In particular, physician empathy and clear communication are associated with higher patient satisfaction and better psychological outcomes, as they help patients feel validated and understood in their experience of symptoms (100). Moreover, patients with PCS or IBS often benefit from a holistic approach that addresses both the physical and psychological components of their conditions, and evidence suggests that improving communication in the clinical setting may also improve adherence to treatment plans and long-term recovery (101). Central to this interaction are advanced communication competencies, including attentive, non-directive listening, empathic validation of the patient’s experience, and the formulation of realistic therapeutic objectives—emphasizing symptom mitigation and functional improvement over curative intent. The strategic use of nonverbal communication—such as sustained eye contact, affirming gestures, forward-leaning posture, and open body orientation—further serves to foster trust and engagement. Critically, the clinical encounter should extend beyond a narrow somatic focus to encompass the patient’s broader biopsychosocial context and individual health-related goals. By fostering trust and ensuring that patients feel heard, physicians can create a supportive environment that enhances the overall quality of care and potentially accelerates recovery.

## Discussion

IBS—especially in its post-infectious form—provides a valuable framework for understanding PCS. Shared features such as immune dysregulation, dysbiosis, autonomic dysfunction, and complex symptomatology suggest that therapeutic strategies used in IBS may be adapted to support PCS patients. The parallels between IBS and PCS highlight the importance of understanding post-infectious syndromes within a biopsychosocial framework. While PCS presents novel challenges, existing models like IBS offer a starting point for developing diagnostic criteria and treatment algorithms. Interdisciplinary collaboration, longitudinal research, and patient engagement are key to advancing care. Establishing a robust therapeutic alliance is a cornerstone in the effective management of patients with IBS, particularly given the complex interplay of biological, psychological, and social factors. The often unsatisfactory outcomes associated with causal therapeutic approaches for IBS support the rationale for symptom-oriented therapeutic approaches. As research advances, integrating insights from chronic functional disorders will be essential to addressing the long-term sequelae of COVID-19 and to rapidly establishing evidence-based treatment pathways for patients affected by PCS.

## References

[1] Lundberg-Morris L Leach S Xu Y Martikainen J Santosa A Gisslén M . Covid-19 vaccine effectiveness against post-covid-19 condition among 589 722 individuals in Sweden: population based cohort study. BMJ. (2023) 383:e076990. doi: 10.1136/bmj-2023-076990, PMID: 37993131 PMC10666099

[2] Nalbandian A Sehgal K Gupta A Madhavan MV McGroder C Stevens JS . Post-acute COVID-19 syndrome. Nat Med. (2021) 27:601–15. doi: 10.1038/s41591-021-01283-z, PMID: 33753937 PMC8893149

[3] Soriano JB Murthy S Marshall JC Relan P Diaz JV WHO Clinical Case Definition Working Group on Post-COVID-19 Conditi;on . A clinical case definition of post-COVID-19 condition by a Delphi consensus. Lancet Infect Dis. (2022) 22:e102–7. doi: 10.1016/S1473-3099(21)00703-9, PMID: 34951953 PMC8691845

[4] Giszas B Trommer S Schüßler N Rodewald A Besteher B Bleidorn J . Post-COVID-19 condition is not only a question of persistent symptoms: structured screening including health-related quality of life reveals two separate clusters of post-COVID. Infection. (2023) 51:365–77. doi: 10.1007/s15010-022-01886-9, PMID: 35869353 PMC9307219

[5] Xie Y Choi T Al-Aly Z . Postacute sequelae of SARS-coV-2 infection in the pre-delta, delta, and omicron eras. N Engl J Med. (2024) 391:515–25. doi: 10.1056/NEJMoa2403211, PMID: 39018527 PMC11687648

[6] Lopez-Leon S Wegman-Ostrosky T Perelman C Sepulveda R Rebolledo PA Cuapio A . More than 50 long-term effects of COVID-19: a systematic review and meta-analysis. Sci Rep. (2021) 11:16144. doi: 10.1038/s41598-021-95565-8, PMID: 34373540 PMC8352980

[7] Stallmach A Kesselmeier M Bauer M Gramlich J Finke K Fischer A . Comparison of fatigue, cognitive dysfunction and psychological disorders in post-COVID patients and patients after sepsis: is there a specific constellation? Infection. (2022) 50:661–9. doi: 10.1007/s15010-021-01733-3, PMID: 34997542 PMC8741139

[8] Taquet M Dercon Q Luciano S Geddes JR Husain M Harrison PJ . Incidence, co-occurrence, and evolution of long-COVID features: A 6-month retrospective cohort study of 273,618 survivors of COVID-19. PLoS Med. (2021) 18:e1003773. doi: 10.1371/journal.pmed.1003773, PMID: 34582441 PMC8478214

[9] Zhang D Chen C Xie Y Zeng F Chen S Chen R . Post-infection functional gastrointestinal disorders following coronavirus disease-19: a prospective follow-up cohort study. BMC Infect Dis. (2023) 23:422. doi: 10.1186/s12879-023-08401-x, PMID: 37344782 PMC10286442

[10] Marasco G Cremon C Barbaro MR Cacciari G Falangone F Kagramanova A . Post COVID-19 irritable bowel syndrome. Gut. (2023) 72:484–92. doi: 10.1136/gutjnl-2022-328483, PMID: 36591612

[11] Canavan C West J Card T . The epidemiology of irritable bowel syndrome. Clin Epidemiol. (2014) 6:71–80. doi: 10.2147/CLEP.S40245, PMID: 24523597 PMC3921083

[12] Lacy BE Xu Y Taylor DCA Kosch KJ Dobrescu R Morlock A . Burden of illness and treatment attitudes among participants meeting Rome IV criteria for irritable bowel syndrome: A nationwide survey in the United States. Neurogastroenterol Motil. (2024) 36:e14903. doi: 10.1111/nmo.14903, PMID: 39223955

[13] Kraimi N Ross T Pujo J De Palma G . The gut microbiome in disorders of gut–brain interaction. Gut Microbes. (2024) 16:2360233. doi: 10.1080/19490976.2024.2360233, PMID: 38949979 PMC11218806

[14] Li J Ghosh TS Arendt E Shanahan F O’Toole PW . Cross-cohort gut microbiome signatures of irritable bowel syndrome presentation and treatment. Adv Sci Weinh Baden-Wurtt Ger. (2024) 11:e2308313. doi: 10.1002/advs.202308313, PMID: 39243395 PMC11538712

[15] Sulaimi F Ong TSK Tang ASP Quek J Pillay RM Low DT . Risk factors for developing irritable bowel syndrome: systematic umbrella review of reviews. BMC Med. (2025) 23:103. doi: 10.1186/s12916-025-03930-5, PMID: 39985070 PMC11846330

[16] Barbara G Grover M Bercik P Corsep M Ghoshal UC Ohman L . Rome foundation working team report on post-infection irritable bowel syndrome. Gastroenterology. (2019) 156:46–58.e7. doi: 10.1053/j.gastro.2018.07.011, PMID: 30009817 PMC6309514

[17] Claessens G Gerritzen I Van Osch F Van Den Bergh JP Verberne D Gach D . Prevalence and predictors of persistent cognitive and psychological symptoms in non-hospitalized post-COVID-19 patients seeking care at an outpatient post-COVID-19 clinic. Front Psychol. (2024) 15:1396963. doi: 10.3389/fpsyg.2024.1396963, PMID: 39193035 PMC11347444

[18] Sudre CH Murray B Varsavsky T Graham MS Penfold RS Bowyer RC . Attributes and predictors of long COVID. Nat Med. (2021) 27:626–31. doi: 10.1038/s41591-021-01292-y, PMID: PMC7611399 33692530

[19] Koczulla AR Ankermann T Behrends U Berlit P Berner R Böing S . German S1 guideline long-/post-COVID. Pneumol Stuttg Ger. (2022) 76:855–907. doi: 10.1055/a-1946-3230, PMID: 36479679

[20] Haunhorst S Dudziak D Scheibenbogen C Seifert M Sotzny F Finke C . Towards an understanding of physical activity-induced post-exertional malaise: Insights into microvascular alterations and immunometabolic interactions in post-COVID condition and myalgic encephalomyelitis/chronic fatigue syndrome. Infection. (2025) 53:1–13. doi: 10.1007/s15010-024-02386-8, PMID: 39240417 PMC11825644

[21] Layer P Andresen V Allescher H Bischoff SC Claßen M Elsenbruch S . Update S3-Leitlinie Reizdarmsyndrom: Definition, Pathophysiologie, Diagnostik und Therapie. Gemeinsame Leitlinie der Deutschen Gesellschaft für Gastroenterologie, Verdauungs- und Stoffwechselkrankheiten (DGVS) und der Deutschen Gesellschaft für Neurogastroenterologie und Motilität (DGNM) – Juni 2021 – AWMF-Registriernummer: 021/016. Z Für Gastroenterol. (2021) 59:1323–415. doi: 10.1055/a-1591-4794, PMID: 34891206

[22] Lembo A Sultan S Chang L Heidelbaugh JJ Smalley W Verne GN . AGA clinical practice guideline on the pharmacological management of irritable bowel syndrome with diarrhea. Gastroenterology. (2022) 163:137–51. doi: 10.1053/j.gastro.2022.04.017, PMID: 35738725

[23] Vasant DH Paine PA Black CJ Houghton LA Everis HA Corsep M . British Society of Gastroenterology guidelines on the management of irritable bowel syndrome. Gut. (2021) 70:1214–40. doi: 10.1136/gutjnl-2021-324598, PMID: 33903147

[24] Giuliano M Tiple D Agostoni P Armocida B Biardi L Bonfigli AR . Italian good practice recommendations on management of persons with Long-COVID. Front Public Health. (2023) 11:1122141. doi: 10.3389/fpubh.2023.1122141, PMID: 37151592 PMC10157646

[25] Koutsiaris AG . A blood supply pathophysiological microcirculatory mechanism for long COVID. Life. (2024) 14:1076. doi: 10.3390/life14091076, PMID: 39337860 PMC11433432

[26] Koutsiaris AG Karakousis K . Long COVID mechanisms, microvascular effects, and evaluation based on incidence. Life. (2025) 15:887. doi: 10.3390/life15060887, PMID: 40566540 PMC12193837

[27] Marshall JK Thabane M Garg AX Clark WF Moayyedi P Collins SM . Eight year prognosis of postinfectious irritable bowel syndrome following waterborne bacterial dysentery. Gut. (2010) 59:605–11. doi: 10.1136/gut.2009.202234, PMID: 20427395

[28] Jung IS Kim HS Park H Lee SI . The clinical course of postinfectious irritable bowel syndrome: a five-year follow-up study. J Clin Gastroenterol. (2009) 43:534–40. doi: 10.1097/MCG.0b013e31818c87d7, PMID: 19262407

[29] Neal KR Barker L Spiller RC . Prognosis in post-infective irritable bowel syndrome: a six year follow up study. Gut. (2002) 51:410–3. doi: 10.1136/gut.51.3.410, PMID: 12171965 PMC1773359

[30] Reuken PA Besteher B Finke K Fischer A Holl A Katzer K . Longterm course of neuropsychological symptoms and ME/CFS after SARS-CoV-2-infection: a prospective registry study. Eur Arch Psychiatry Clin Neurosci. (2024) 274:1903–10. doi: 10.1007/s00406-023-01661-3, PMID: 37587244 PMC11579079

[31] Lemhöfer C Sturm C Loudovici-Krug D Guntenbrunner C Bülow M Reuken P . Quality of life and ability to work of patients with Post-COVID syndrome in relation to the number of existing symptoms and the duration since infection up to 12 months: a cross-sectional study. Qual Life Res Int J Qual Life Asp Treat Care Rehabil. (2023) 32:1991–2002. doi: 10.1007/s11136-023-03369-2, PMID: 36869248 PMC9984128

[32] Marshall JK Thabane M Garg AX Clark WF Salvadori M Collins SM . Incidence and epidemiology of irritable bowel syndrome after a large waterborne outbreak of bacterial dysentery. Gastroenterology. (2006) 131:445–450; quiz 660. doi: 10.1053/j.gastro.2006.05.053, PMID: 16890598

[33] Spiller R Garsed K . Postinfectious irritable bowel syndrome. Gastroenterology. (2009) 136:1979–88. doi: 10.1053/j.gastro.2009.02.074, PMID: 19457422

[34] Gwee K-A Collins SM Read NW Rajnakova A Deng Y Graham JC . Increased rectal mucosal expression of interleukin 1beta in recently acquired post-infectious irritable bowel syndrome. Gut. (2003) 52:523–6. doi: 10.1136/gut.52.4.523, PMID: 12631663 PMC1773606

[35] Tilikete C Zamali I Meddeb Z Kharroubi G Marzouki S Dhaouadi T . Exploring the landscape of symptom-specific inflammatory cytokines in post-COVID syndrome patients. BMC Infect Dis. (2024) 24:1337. doi: 10.1186/s12879-024-10222-5, PMID: 39578766 PMC11583569

[36] Cryan JF O’Riordan KJ Cowan CSM Sandhu KV Bastiaanssen TFS Boehme M . The microbiota-gut-brain axis. Physiol Rev. (2019) 99:1877–2013. doi: 10.1152/physrev.00018.2018, PMID: 31460832

[37] Livanos AE Jha D Cossarini F Gonzalez-Reiche AS Tokuyama M Aydillo T . Intestinal host response to SARS-coV-2 infection and COVID-19 outcomes in patients with gastrointestinal symptoms. Gastroenterology. (2021) 160:2435–2450.e34. doi: 10.1053/j.gastro.2021.02.056, PMID: 33676971 PMC7931673

[38] Su Y Yuan D Chen DG Ng RH Wang K Choi J . Multiple early factors anticipate post-acute COVID-19 sequelae. Cell. (2022) 185:881–895.e20. doi: 10.1016/j.cell.2022.01.014, PMID: 35216672 PMC8786632

[39] Phetsouphanh C Darley DR Wilson DB Howe A Munier CML Patel SK . Immunological dysfunction persists for 8 months following initial mild-to-moderate SARS-CoV-2 infection. Nat Immunol. (2022) 23:210–6. doi: 10.1038/s41590-021-01113-x, PMID: 35027728

[40] Yu LC-H . Gastrointestinal pathophysiology in long COVID: Exploring roles of microbiota dysbiosis and serotonin dysregulation in post-infectious bowel symptoms. Life Sci. (2024) 358:123153. doi: 10.1016/j.lfs.2024.123153, PMID: 39454992

[41] Chadwick VS Chen W Shu D Paulus B Bethwaite P Tie A . Activation of the mucosal immune system in irritable bowel syndrome. Gastroenterology. (2002) 122:1778–83. doi: 10.1053/gast.2002.33579, PMID: 12055584

[42] Dunlop SP Hebden J Campbell E Naesdal J Olbe L Perkins AC . Abnormal intestinal permeability in subgroups of diarrhea-predominant irritable bowel syndromes. Am J Gastroenterol. (2006) 101:1288–94. doi: 10.1111/j.1572-0241.2006.00672.x, PMID: 16771951

[43] Andus I Büttner J Bochow-Fitzner B Tacke F Jochum C . Intestinal permeability correlated with chronic fatigue in a patient with long COVID—A case report and overview of the literature. Front Med. (2026) 13:1725242. doi: 10.3389/fmed.2026.1725242, PMID: 41704685 PMC12907148

[44] Mitselou A Grammeniatis V Varouktsi A Papadatos SS Katsanos K Galani V . Proinflammatory cytokines in irritable bowel syndrome: a comparison with inflammatory bowel disease. Intest Res. (2020) 18:115–20. doi: 10.5217/ir.2019.00125, PMID: PMC7000645 32013318

[45] Dinan TG Quigley EMM Ahmed SMM Scully P O’Brien S O’Mahony L . Hypothalamic-pituitary-gut axis dysregulation in irritable bowel syndrome: plasma cytokines as a potential biomarker? Gastroenterology. (2006) 130:304–11. doi: 10.1053/j.gastro.2005.11.033, PMID: 16472586

[46] Bergantini L Gangi S d’Alessandro M Cameli P Perea B Meocci M . Altered serum concentrations of IL-8, IL-32 and IL-10 in patients with lung impairment 6 months after COVID-19. Immunobiology. (2024) 229:152813. doi: 10.1016/j.imbio.2024.152813, PMID: 38805808

[47] Schultheiß C Willscher E Paschold L Gosschick C Klee B Henkes S-S . The IL-1β, IL-6, and TNF cytokine triad is associated with post-acute sequelae of COVID-19. Cell Rep Med. (2022) 3:100663. doi: 10.1016/j.xcrm.2022.100663, PMID: 35732153 PMC9214726

[48] Vara EJ Brokstad K Hausken T Lied GA . Altered levels of cytokines in patients with irritable bowel syndrome are not correlated with fatigue. Int J Gen Med. (2018) 11:285–91. doi: 10.2147/IJGM.S166600, PMID: 30013383 PMC6038856

[49] Haunhorst S Bloch W Javelle F Krüger K Baumgart S Drube S . A scoping review of regulatory T cell dynamics in convalescent COVID-19 patients – indications for their potential involvement in the development of Long COVID? Front Immunol. (2022) 13:1070994. doi: 10.3389/fimmu.2022.1070994, PMID: 36582234 PMC9792979

[50] Öhman L Isaksson S Lindmark A-C Posserud I Stotzer P-O Strid H . T-cell activation in patients with irritable bowel syndrome. Am J Gastroenterol. (2009) 104:1205–12. doi: 10.1038/ajg.2009.116, PMID: 19367268

[51] Sumantri S Rengganis I . Immunological dysfunction and mast cell activation syndrome in long COVID. Asia Pac Allergy. (2023) 13:50–3. doi: 10.5415/apallergy.0000000000000022, PMID: 37389095 PMC10166245

[52] Weinstock LB Brook JB Walters AS Goris A Afrin LB Molderings GJ . Mast cell activation symptoms are prevalent in Long-COVID. Int J Infect Dis. (2021) 112:217–26. doi: 10.1016/j.ijid.2021.09.043, PMID: 34563706 PMC8459548

[53] Barbara G Stanghellini V De Giorgio R Cremon C Cosrell GS Santini D . Activated mast cells in proximity to colonic nerves correlate with abdominal pain in irritable bowel syndrome. Gastroenterology. (2004) 126:693–702. doi: 10.1053/j.gastro.2003.11.055, PMID: 14988823

[54] Mehta P McAuley DF Brown M Sanchez E Tasersall RS Manson JJ . COVID-19: consider cytokine storm syndromes and immunosuppression. Lancet. (2020) 395:1033–4. doi: 10.1016/S0140-6736(20)30628-0, PMID: 32192578 PMC7270045

[55] Ng QX Soh AYS Loke W Lim DY Yeo W-S . The role of inflammation in irritable bowel syndrome (IBS). J Inflammation Res. (2018) 11:345–9. doi: 10.2147/JIR.S174982, PMID: 30288077 PMC6159811

[56] Hattori F Nishiyama J Hasuo H . Correlation of interferons and autoimmune aspects in long COVID-19 patients. Int Immunol. (2025) 37:355–63. doi: 10.1093/intimm/dxaf008, PMID: 39921694 PMC12096164

[57] Gupta G Buonsenso D Wood J Mohandas S Warburton D . Mechanistic insights into long covid: viral persistence, immune dysregulation, and multi-organ dysfunction. Compr Physiol. (2025) 15:e70019. doi: 10.1002/cph4.70019, PMID: 40474772

[58] Kasti A Katsas K Nikolaki MD Triantafyllou K . The role and the regulation of NLRP3 inflammasome in irritable bowel syndrome: A narrative review. Microorganisms. (2025) 13:171. doi: 10.3390/microorganisms13010171, PMID: 39858939 PMC11767632

[59] Belmonte L Beutheu Youmba S Bertiaux-Vandaële N Antoniep M Lecleire S Zalar A . Role of toll like receptors in irritable bowel syndrome: differential mucosal immune activation according to the disease subtype. PLoS One. (2012) 7:e42777. doi: 10.1371/journal.pone.0042777, PMID: 23028414 PMC3461726

[60] Zhao J Dai Y Tian J Lei L Zhou X . Impact of mechanical barrier damage and interleukin-17 on symptoms in patients with post-infectious irritable bowel syndrome. Br J Hosp Med. (2024) 85:1–13. doi: 10.12968/hmed.2024.0114, PMID: 39078895

[61] Meynier M Baudu E Rolhion N Defaye M Straube M Daugey V . AhR/IL-22 pathway as new target for the treatment of post-infectious irritable bowel syndrome symptoms. Gut Microbes. (2022) 14:2022997. doi: 10.1080/19490976.2021.2022997, PMID: 35090380 PMC8803069

[62] Chen X Cunha Carvalho B Sinha A Pisman N Benkov K Lai J . Irritable bowel syndrome with diarrhea in pediatric patients is associated with type 2 and type 9 T cells in the intestinal mucosa. Cell Mol Gastroenterol Hepatol. (2025) 19:101488. doi: 10.1016/j.jcmgh.2025.101488, PMID: 40024536 PMC12264213

[63] Liu Q Mak JWY Su Q Yeoh YK Lui GC-Y Ng SSS . Gut microbiota dynamics in a prospective cohort of patients with post-acute COVID-19 syndrome. Gut. (2022) 71:544–52. doi: 10.1136/gutjnl-2021-325989, PMID: 35082169

[64] Iqbal NT Khan H Khalid A Mahmood SF Nasir N Khanum I . Chronic inflammation in post-acute sequelae of COVID-19 modulates gut microbiome: a review of literature on COVID-19 sequelae and gut dysbiosis. Mol Med Camb Mass. (2025) 31:22. doi: 10.1186/s10020-024-00986-6, PMID: 39849406 PMC11756069

[65] Lau RI Su Q Ng SC . Long COVID and gut microbiome: insights into pathogenesis and therapeutics. Gut Microbes. (2025) 17:2457495. doi: 10.1080/19490976.2025.2457495, PMID: 39854158 PMC11776476

[66] Righi E Dalla Vecchia I Auerbach N Morra M Górska A Sciammarella C . Gut microbiome disruption following SARS-coV-2: A review. Microorganisms. (2024) 12:131. doi: 10.3390/microorganisms12010131, PMID: 38257958 PMC10820238

[67] Lee L-H Law JW-F Tan LT-H Letchumanan V Lim HX . IDDF2024-ABS-0299 Gut microbiota changes in relation to long-COVID-19 syndrome. In: Basic gastroenterology. London, UK: BMJ Publishing Group Ltd and British Society of Gastroenterology (2024). p. A192–2.

[68] Ferreira-Junior AS Borgonovi TF De Salis LVV Leite AZ Dantas AS De Salis GVV . Detection of intestinal dysbiosis in post-COVID-19 patients one to eight months after acute disease resolution. Int J Environ Res Public Health. (2022) 19:10189. doi: 10.3390/ijerph191610189, PMID: 36011823 PMC9408204

[69] Vestad B Ueland T Lerum TV Dahl TB Holm K Barras-Due A . Respiratory dysfunction three months after severe COVID-19 is associated with gut microbiota alterations. J Intern Med. (2022) 291:801–12. doi: 10.1111/joim.13458, PMID: 35212063 PMC9115297

[70] An Y He L Xu X Piao M Wang B Liu T . Gut microbiota in post-acute COVID-19 syndrome: not the end of the story. Front Microbiol. (2024) 15:1500890. doi: 10.3389/fmicb.2024.1500890, PMID: 39777148 PMC11703812

[71] Bistagnino F Pizzi D Mantovani F Antonino JR Tovani-Palone MR . Long COVID and gut candidiasis: What is the existing relationship? World J Gastroenterol. (2024) 30:4104–14. doi: 10.3748/wjg.v30.i37.4104, PMID: 39474404 PMC11514539

[72] Andresen V Layer P . Irritable bowel syndrome - a disease. Dtsch Med Wochenschr 1946. (2018) 143:411–9. doi: 10.1055/s-0043-125224, PMID: 29544237

[73] Napolitano M Fasulo E Ungaro F Massimino L Sinagra E Danese S . Gut dysbiosis in irritable bowel syndrome: A narrative review on correlation with disease subtypes and novel therapeutic implications. Microorganisms. (2023) 11:2369. doi: 10.3390/microorganisms11102369, PMID: 37894027 PMC10609453

[74] Pittayanon R Lau JT Yuan Y Leontiadis GI Tse F Surese M . Gut microbiota in patients with irritable bowel syndrome-A systematic review. Gastroenterology. (2019) 157:97–108. doi: 10.1053/j.gastro.2019.03.049, PMID: 30940523

[75] Arruda IDSA Cavalcante CDS Rubens RS Castro LNPDF Nóbrega YKDM Dalmolin TV . Changes in the gut microbiota of patients after SARS-coV-2 infection: what do we know? Microorganisms. (2025) 13:2529. doi: 10.3390/microorganisms13112529, PMID: 41304215 PMC12654389

[76] Shrestha B Patel D Shah H Hanna KS Kaur H Alazzeh MS . The role of gut-microbiota in the pathophysiology and therapy of irritable bowel syndrome: A systematic review. Cureus. (2022) 14(8):e28064. doi: 10.7759/cureus.28064, PMID: 36127988 PMC9477602

[77] Alpert O Begun L Garren P Solhkhah R . Cytokine storm induced new onset depression in patients with COVID-19. A new look into the association between depression and cytokines -two case reports. Brain Behav Immun - Health. (2020) 9:100173. doi: 10.1016/j.bbih.2020.100173, PMID: 33163979 PMC7606074

[78] Lorkiewicz P Waszkiewicz N . Biomarkers of post-COVID depression. J Clin Med. (2021) 10:4142. doi: 10.3390/jcm10184142, PMID: 34575258 PMC8470902

[79] Penninx BWJH . Psychiatric symptoms and cognitive impairment in “Long COVID”: the relevance of immunopsychiatry. World Psychiatry. (2021) 20:357–8. doi: 10.1002/wps.20913, PMID: 34505378 PMC8429338

[80] Hirschenberger M Hunszinger V Sparrer KMJ . Implications of innate immunity in post-acute sequelae of non-persistent viral infections. Cells. (2021) 10:2134. doi: 10.3390/cells10082134, PMID: 34440903 PMC8391718

[81] Maltezou HC Pavli A Tsakris A . Post-COVID syndrome: an insight on its pathogenesis. Vaccines. (2021) 9:497. doi: 10.3390/vaccines9050497, PMID: 34066007 PMC8151752

[82] Dani M Dirksen A Taraborrelli P Torocastro M Panagopoulos D Suson R . Autonomic dysfunction in “long COVID”: rationale, physiology and management strategies. Clin Med Lond Engl. (2021) 21:e63–7. doi: 10.7861/clinmed.2020-0896, PMID: 33243837 PMC7850225

[83] Choutka J Jansari V Hornig M Iwasaki A . Unexplained post-acute infection syndromes. Nat Med. (2022) 28:911–23. doi: 10.1038/s41591-022-01810-6, PMID: 35585196

[84] Chu L Valencia IJ Garvert DW Montoya JG . Deconstructing post-exertional malaise in myalgic encephalomyelitis/chronic fatigue syndrome: A patient-centered, cross-sectional survey. PLoS One. (2018) 13:e0197811. doi: 10.1371/journal.pone.0197811, PMID: 29856774 PMC5983853

[85] Yildirim AE Korkmaz M Altun R Sandikçi SC Ocal S Selçuk H . Is there any association between irritable bowel syndrome subgroups and autonomous dysfunction. Eur Rev Med Pharmacol Sci. (2016) 20:1315–22. 27097952

[86] Manabe N Tanaka T Hata J Kusunoki H Haruma K . Pathophysiology underlying irritable bowel syndrome--from the viewpoint of dysfunction of autonomic nervous system activity. J Smooth Muscle Res Nihon Heikatsukin Gakkai Kikanshi. (2009) 45:15–23. doi: 10.1540/jsmr.45.15, PMID: 19377269

[87] Salvioli B Pellegatta G Malacarne M Pace F Malesci A Pagani M . Autonomic nervous system dysregulation in irritable bowel syndrome. Neurogastroenterol Motil. (2015) 27:423–30. doi: 10.1111/nmo.12512, PMID: 25581440

[88] Liu Q Wang EM Yan XJ Chen SL . Autonomic functioning in irritable bowel syndrome measured by heart rate variability: a meta-analysis. J Dig Dis. (2013) 14:638–46. doi: 10.1111/1751-2980.12092, PMID: 23927739

[89] Ranisavljev M Stajer V Todorovic N Ostojic J Cvejic JH Steinert RE . The effects of 3-month supplementation with synbiotic on patient-reported outcomes, exercise tolerance, and brain and muscle metabolism in adult patients with post-COVID-19 chronic fatigue syndrome (STOP-FATIGUE): a randomized Placebo-controlled clinical trial. Eur J Nutr. (2024) 64:28. doi: 10.1007/s00394-024-03546-0, PMID: 39592468

[90] Carpallo-Porcar B Jiménez-Sánchez C Calvo S Irún P Kolesnyk-Sumskaya E AllerBlanco AI . ARACOV-02. Specialized nutritional intervention and telerehabilitation in patients with long COVID: Protocol of a randomized controlled trial. PLoS One. (2025) 20:e0321811. doi: 10.1371/journal.pone.0321811, PMID: 40299883 PMC12040102

[91] Staudacher HM Whelan K Irving PM Lomer MCE . Comparison of symptom response following advice for a diet low in fermentable carbohydrates (FODMAPs) versus standard dietary advice in patients with irritable bowel syndrome. J Hum Nutr Diet. (2011) 24:487–95. doi: 10.1111/j.1365-277X.2011.01162.x, PMID: 21615553

[92] Bigman G Rusu ME Shelawala N Sorkin JD Beamer BA Ryan AS . A comprehensive scoping review on diet and nutrition in relation to long COVID-19 symptoms and recovery. Nutrients. (2025) 17:1802. doi: 10.3390/nu17111802, PMID: 40507071 PMC12157204

[93] Barrea L Vetrani C Caprio M Cataldi M Ghoch ME Elce A . From the ketogenic diet to the mediterranean diet: the potential dietary therapy in patients with obesity after coVID-19 infection (Post coVID syndrome). Curr Obes Rep. (2022) 11:144–65. doi: 10.1007/s13679-022-00475-z, PMID: 35524067 PMC9075143

[94] Naureen Z Dautaj A Nodari S Fiorep F Dhuli K Anpilogov K . Proposal of a food supplement for the management of post-COVID syndrome. Eur Rev Med Pharmacol Sci. (2021) 25:67–73. doi: 10.26355/eurrev_202112_27335, PMID: 34890036

[95] Cheong K-L Yu B Teng B Veeraperumal S Xu B Zhong S . Post-COVID-19 syndrome management: Utilizing the potential of dietary polysaccharides. BioMed Pharmacother. (2023) 166:115320. doi: 10.1016/j.biopha.2023.115320, PMID: 37595427

[96] Ford AC Lacy BE Harris LA Quigley EMM Moayyedi P . Effect of antidepressants and psychological therapies in irritable bowel syndrome: an updated systematic review and meta-analysis. Am J Gastroenterol. (2019) 114:21–39. doi: 10.1038/s41395-018-0222-5, PMID: 30177784

[97] Greene C Connolly R Brennan D Laffan A O’Keeffe E Zaporojan L . Blood–brain barrier disruption and sustained systemic inflammation in individuals with long COVID-associated cognitive impairment. Nat Neurosci. (2024) 27:421–32. doi: 10.1038/s41593-024-01576-9, PMID: 38388736 PMC10917679

[98] Kempuraj D Aenlle KK Cohen J Mathew A Isler D Pangeni RP . COVID-19 and long COVID: disruption of the neurovascular unit, blood-brain barrier, and tight junctions. Neuroscientist. (2024) 30:421–39. doi: 10.1177/10738584231194927, PMID: 37694571

[99] Davis HE McCorkell L Vogel JM Topol EJ . Long COVID: major findings, mechanisms and recommendations. Nat Rev Microbiol. (2023) 21:133–46. doi: 10.1038/s41579-022-00846-2, PMID: 36639608 PMC9839201

[100] Al-Zyoud W Oweis T Al-Thawabih H Al-Saqqar F Al-Kazwini A Al-Hammouri F . The psychological effects of physicians’ Communication skills on COVID-19 patients. Patient Prefer Adherence. (2021) 15:677–90. doi: 10.2147/PPA.S303869, PMID: 33854302 PMC8039207

[101] Reuken PA Trommer S Besteher B Bleidorn J Finke K Lemhöfer C . Outpatient long/post-COVID care: barriers and desires of affected persons to medical care. Gesundheitswes Bundesverb Arzte. (2023) 85:1072–5. doi: 10.1055/a-2035-9431, PMID: 37142235 PMC11248071

